# Enabling Electrochemical–Mechanical Robustness of Ultra‐High Ni Cathode via Self‐Supported Primary‐Grain‐Alignment Strategy

**DOI:** 10.1002/advs.202306347

**Published:** 2023-10-26

**Authors:** Yu‐Kun Hou, Chenxi Li, Dongsheng Ren, Feixiong He, Kaijun Zhuang, Shuo Yin, Guohe Yuan, Yiqiao Wang, Yi Guo, Saiyue Liu, Peng Sun, Zhihua Zhang, Tiening Tan, Gaolong Zhu, Languang Lu, Xiang Liu, Minggao Ouyang

**Affiliations:** ^1^ School of Vehicle and Mobility Tsinghua University Beijing 100084 China; ^2^ Prof. Ouyang Minggao Academician Workstation Sichuan new Energy Vehicle innovation Center Co., Ltd. Yibin 644000 China; ^3^ School of Materials Science and Engineering Beihang University Beijing 100191 China; ^4^ School of Control and Computer Engineering North China Electric Power University Beijing 102208 China; ^5^ CNGR advanced material Co., Ltd. Tongren 554000 China; ^6^ Changzhou Institute of Advanced Manufacturing Technology 213000 Changzhou China

**Keywords:** cathode designs, electrochemical–mechanical properties, Ni‐rich cathodes, Lithium ion batteries

## Abstract

The electrochemical–mechanical degradation of ultrahigh Ni cathode for lithium‐ion batteries is a crucial aspect that limits the cycle life and safety of devices. Herein, the study reports a facile strategy involving rational design of primary grain crystallographic orientation within polycrystalline cathode, which well enhanced its electro‐mechanical strength and Li^+^ transfer kinetics. Ex situ and in situ experiments/simulations including cross‐sectional particle electron backscatter diffraction (EBSD), single‐particle micro‐compression, thermogravimetric analysis combined with mass spectrometry (TGA‐MS), and finite element modeling reveal that, the primary‐grain‐alignment strategy effectively mitigates the particle pulverization, lattice oxygen release thereby enhances battery cycle life and safety. Besides the preexisting doping and coating methodologies to improve the stability of Ni‐rich cathode, the primary‐grain‐alignment strategy, with no foreign elements or heterophase layers, is unprecedently proposed here. The results shed new light on the study of electrochemical–mechanical strain alleviation for electrode materials.

## Graphical abstract

In this work, to boost the electrochemical–mechanical robustness of ultrahigh Ni cathode, without resorting to any taxing processes of foreign elements doping and surface heterophase coating, a facile self‐supported primary‐grain‐alignment strategy involving reasonable engineering of primary grain crystallographic orientation within polycrystalline cathode is unprecedently proposed. This work sheds new light on the study of electrochemical–mechanical strain alleviation for electrode materials.

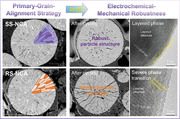

## 1 Introduction

Currently, the development of clean, efficient, and safe energy storage technologies is crucial in achieving global Carbon Neutrality goals.^[^ ^1^ ^]^ Among those, the lithium‐ion battery, take the first place as energy storage devices for portable devices and electric vehicles, due to its superior energy density, rate capability, cycle life and cost efficiency compared to other energy storage technologies.^[^ ^2^, ^3^, ^4^ ^]^ However, the growing requirement for an higher energy density LIBs calls for rapid revolution of the battery materials.^[^ ^4^, ^5^ ^]^ Among, the cathode material is recognized as the bottleneck that restricts the battery energy density and safety.^[^ ^5^, ^6^ ^]^ Up‐to‐date, the polycrystalline ternary Li[Ni_x_Co_y_(Al or Mn)_1−x−y_]O_2_ (NCA/NCM) demonstrates the most state‐of‐the‐art cathodes for high energy density commercial LIBs.^[^ ^7^, ^8^ ^]^ While, continuous increasing the nickel content of NCM/NCA beyond 90%, is identified to be one of the main battery material roadmap with larger capacity and higher voltage.^[^ ^9^ ^]^ However, the increase of Ni content in NCM/NCA can cause structure instability due to irreversible cation migrations and oxygen evolutions with excessive lithium utilization, therefore leads to a fast capacity fading,^[^ ^10^, ^11^, ^12^, ^13^, ^14^ ^]^ and even thermal runaway safety issues.^[^ ^10^, ^11^ ^]^

Enormous efforts have been made to modify the structural instability of Ni‐rich layered oxides cathode.^[^ ^8^, ^15^, ^16^, ^17^, ^18^, ^19^, ^20^, ^21^, ^22^, ^23^, ^24^ ^]^ For example, the compositions of Ni‐rich NCA and NCM oxides can be tuned by foreign elements doping with the pillar effect (Zr, Nb, Ti, Mo, Ta, W, etc.).^[^ ^15^, ^16^, ^17^, ^18^ ^]^ However, the doped inactive ingredients sacrifice the specific capacity of the active materials, and more seriously, foreign elements also tend to occupy the ion diffusion channels resulting in sluggish Li‐ion transfer kinetics.^[^ ^18^, ^19^ ^]^ Concurrently, the surface coatings (TiO_2_, ZnO, Al_2_O_3_, Li_3_PO_4_, AlF_3_, etc.) have been introduced on both secondary and primary particles to avoid the deleterious reactions between the cathode and electrolyte.^[^ ^20^, ^21^, ^22^, ^23^ ^]^ Whereas, the mismatched crystalline interface between the host cathode and coating components may detached upon cycling by accumulated cathode lattice strain.^[^ ^23^, ^24^ ^]^ Both coating and doping technologies advocated the commercialization of NCM/NCA cathodes yet failed to stabilize the ultrahigh Ni cathodes.

It is believed that the stress‐strains buildup between the crystal grains, accompanying with the anisotropic lattice unit‐cell evolution during the charge/discharge process, known as the electrochemical–mechanical failure, is one main reason lead for intragranular/intergranular microcracks and aggregate‐structure pulverization of ultrahigh Ni cathode.^[^ ^15^, ^25^, ^26^, ^27^ ^]^ Hence, enhancing the electrochemical–mechanical robustness of ultrahigh Ni cathode is the key, however has long been untouched.^[^ ^28^, ^29^, ^30^ ^]^ Here in this work, without introducing any foreign doping element or surface coating layer, we unprecedently developed a primary‐grain‐alignment strategy with breathable surface‐shield surface to boost the electrochemical–mechanical stability of ultrahigh Ni cathode.

Namely, a surface‐shielded NCA (SS‐NCA) polycrystalline LiNi_0.90_Co_0.08_Al_0.02_O_2_ cathode, with the radially‐oriented core grains, which are conducive to promoting Li^+^ transmission with along crystallographic (003) planes,^[^ ^15^, ^28^ ^]^ more importantly, the cathode outer rim is constituted by densely packed randomly‐oriented grains with much smaller grain size with the same composition, in favor of suppressing intrinsic microcracks propagating from radially‐aligned grains to the surface,^[^ ^24^, ^25^, ^26^, ^27^ ^]^ thereby reinforcing the electromechanics of the active particle. To validate the superiority of this tactical SS‐NCA, a contrast polycrystalline NCA consisting of entirely radially‐oriented grains with equal composition and secondary‐particle size is prepared, which is noted as radial‐structured NCA (RS‐NCA). As is systematically investigated in this study, the surface‐shielded NCA cathode delivers excellent performance in terms of mechanical strength, capacity retention, rate capability, and thermal safety for mitigating oxygen release. Hereby, our work sheds new light on enhancing the electrochemical and structure stability of Ni‐rich layered oxide cathodes through strategic engineering of grain orientation and inter‐assemblies for high‐performance lithium‐ion batteries.

## 2 Results and Discussion

### 2.1 Microstructure and Grains Arrangement Characterization

**Figure 1:**
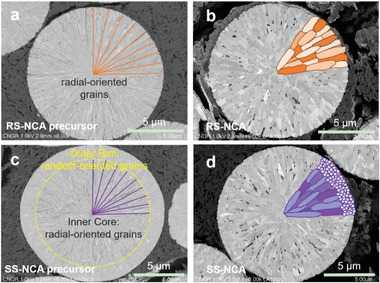
Morphological features of the precursors and cathode materials of RS‐NCA and SS‐NCA. SEM image of a cross‐sectional precursor particle and the corresponding schematic of the primary grains for the a) RS‐NCA and c) SS‐NCA. SEM image of a cross‐sectional cathode particle and the corresponding schematic of the primary grains for the b) RS‐NCA and d) SS‐NCA.

**Figure 2:**
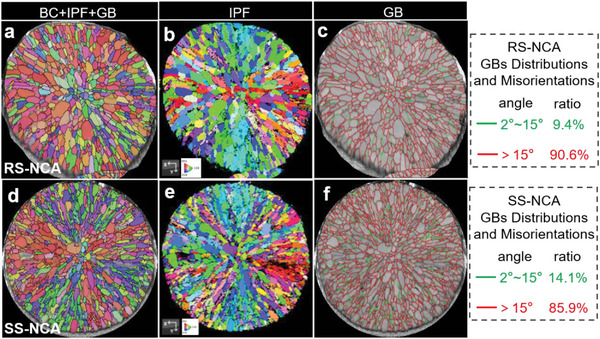
EBSD characterization of internal crystallographic domains of the SS‐NCA and RS‐NCA. Cathode particle cross‐section analyses with EBSD provided primary‐grain arrangement of a) RS‐NCA and d) SS‐NCA, b) crystal orientations of RS‐NCA and e) SS‐NCA, and GB distributions and misorientations of c) RS‐NCA and f) SS‐NCA, respectively. BC, IPF, and GB refer to Band Contrast, Inverse Pole Figure map, and Grain Boundary, respectively.

**Figure** **1** and Figure S1 (Supporting Information), summarized the morphological primary grain alignment of the precursors and cathode materials of RS‐NCA and SS‐NCA. The primary‐grain‐alignment is inherent from the precursor design with carefully tuned coprecipitation condition, see experimental details. As shown in Figure 1a, the primary grain of RS‐NCA precursor arranged entirely radially from center. In contrast, only the core grains of the SS‐NCA precursor are arranged in a radial pattern, while the outer grains exhibit random arrangement, as illustrated in Figure 1c. With the following lithiation and sintering treatment, the RS‐NCA (Figure 1b) and SS‐NCA (Figure 1d) cathode materials are obtained with well‐inherited morphological features of their corresponding grain‐arranged precursors. The Ni, Co, Al, O elements are fully distributed on the as‐prepared cathode particles (see Figure S2, Supporting Information), and the phase of the as‐prepared cathodes are in good match with the typical layered structure of *α*‐NaFeO_2_ (see Figure S3, Supporting Information). Previous studies have indicated that the radial‐oriented grains growing along crystalline *a–b* planes can orientate the Li slabs to point outward from the particle center, thereby facilitating rapid Li diffusion of the active particles.^[^ ^28^, ^31^ ^]^ Furthermore, Sun et al., suggested that the circumferential strain between radial‐oriented grains effectively suppresses local stress concentrations and enables individual grains to uniformly contract and expand during cycling, thereby minimizing local strain build‐up and intergranular microcracking.^[^ ^15^, ^25^, ^26^, ^31^ ^]^ However, in practical preparation of high Ni cathode, the intragranular/intergranular defects and orientation angular mismatches among radial grains can easily lead to stress‐strains cumulation along radial direction boundaries, making it a challenging task to achieve the desired structural stability. ^27^, ^28^, ^31^, ^32^ ^]^ In consequence, detrimental cracks outspread along the radial RS‐NCA particle from surface to center, for both precursor and oxide cathode, were observed as depicted in Figures S1c and S4 (Supporting Information). By comparison, through primary‐grain‐alignment modification with a protective shell of densely packed random‐oriented grains, no microcracks were observed on the surface of the modified SS‐NCA cathode particles (see Figures S1f and S5, Supporting Information). Furthermore, electron backscatter diffraction (EBSD) technique was used to characterize the internal crystallographic domains of the SS‐NCA and RS‐NCA cathode particles, including the primary grain orientations (**Figure** **2**a,b,d,e), grain boundaries (GBs), and boundary misorientation distributions (Figure 2c,f). The EBSD technique is capable of accurately analyzing the grain orientation distributions, GB misorientation distributions, phase distributions, and morphological features of electrode particles (see Figures S6 and S7, Supporting Information).^[^ ^33^, ^34^ ^]^ It can be observed in Figure 2a,b,d,e that the arrangement of radial grains inside the particle is not in perfect circumferential forms, and existing intergranular cavities and angular discordances between radial grains, providing opportunities for microcrack propagation during sintering and charging/discharging of cathode particles.^[^ ^15^, ^25^, ^26^, ^27^, ^31^ ^]^ Therefore, to achieve superior structural and electromechanical stability of the NCA cathode, it is imperative to implement strength‐enhancing modifications on the conventionally prepared radial‐structured NCA particles.^[^ ^15^, ^26^ ^]^ Besides, since Li ions transmit through tortuous paths by connecting the Li‐containing *a*–*b* planes of each grain, the synergetic orientation arrangement of grains plays a crucial role in facilitating easier and faster Li^+^ conduction between cooperating grains.^[^ ^34^ ^]^ Typically, a small misorientation angle at grain boundaries indicates facile inter‐grain Li transfer, whereas a large misorientation angle suggests hindered Li‐ion migration between grains.^[^ ^33^, ^34^, ^35^ ^]^ Based on the EBSD grain boundary misorientation testing (as shown in Figure 2c,f), the red‐colored GBs were identified as large‐angle misoriented GBs (LAMGBs, angle>15°) with impeded Li^+^ transfer kinetics, and the green‐colored GBs were recognized as small‐angle misoriented GBs (SAMGBs, angle between 2° and 15°) with easy Li^+^ transfer kinetics.^[^ ^33^, ^34^, ^35^ ^]^ As a result, the GBs proportion of SAMGBs in SS‐NCA (14.1%) is significantly higher than that in RS‐NCA (9.43%), implying an enhanced and accelerated Li‐ion transport kinetics within the SS‐NCA cathode particles.^[^ ^34^, ^35^ ^]^

### 2.2 Mechanical–Electrochemical Stability Characterization

**Figure 3:**
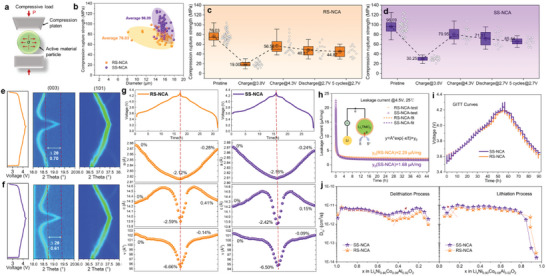
Mechanical and electrochemical properties of RS‐NCA and SS‐NCA. a) Schematic diagram of single‐particle micro‐compression test. b) Compression rupture strengths and average particle hardness of stochastic 50 particles for RS‐NCA and SS‐NCA, respectively. Boxplots of compression rupture strengths of stochastic particles for c) RS‐NCA and d) SS‐NCA at various electrochemical states: pristine, charged at 3.8 V, charged at 4.3 V, discharged at 2.7 V, and after C/10 five cycles discharged at 2.7 V, respectively. In situ synchrotron XRD of e) RS‐NCA and f) SS‐NCA cathodes during charge/discharge within 2.7–4.3 V at C/10. g) The variation of lattice parameters (*a*, *c*, and unit‐cell volume *v*) of RS‐NCA and SS‐NCA cathodes during charge/discharge within 2.7‐4.3 V. h) Parasitic reaction study of RS‐NCA and SS‐NCA cathodes with typical current relaxation curves collected to extract the static leakage current at 4.5 V. S and S^+^ in (h) represent solvents and oxidized solvents, respectively. i) GITT curves of RS‐NCA and SS‐NCA cathodes with pulsed current of 0.3C charging/discharging for 10 min followed by 2 h rest to reach the equilibrium state. j) Li^+^ diffusion coefficients (*D*_Li+_) of RS‐NCA and SS‐NCA cathodes as a function of lithium content in Li_x_Ni_0.90_Co_0.08_Al_0.02_O_2_ during charging and discharging.

To compare the mechanical properties of the SS‐NCA and RS‐NCA cathode, single‐particle micro‐compression rupture strength tests (see **Figure** **3**a) were exclusively performed for both fresh and cycled cathodes. The hardness of the cathode particles was determined by their compression rupture strength (*H*), which is calculated at the breaking point using the applied compressive force (*P*) and particle diameter (*r*) according to equation of *H* = 2.8P/πr^2^ proposed by Oka et al.^[^ ^36^, ^37^ ^]^ As shown in Figure 3b, each 50 different particles at a similar particle size is individually characterized for both RS‐NCA and SS‐NCA cathode, and the compression rupture strength is statistically summarized, for average 76.03 MPa with RS‐NCA and 96.09 MPa with SS‐NCA cathode, indicating that the particle hardness of SS‐NCA is enhanced by 26% compared to that of RS‐NCA due to the optimized grain arrangement (the typical tested compression force‐displacement curves are depicted in Figure S8, Supporting Information). In order to further evaluate the mechanical robustness of cathode particles in an electrochemical active state, we conducted ex situ micro‐compression tests on SS‐NCA and RS‐NCA cathode particles at various charging states and cycles. The different electrochemical states are shown in Figure S9 (Supporting Information), namely charged at 3.8, 4.3 V then discharge at 2.7 V for the 1st and 5th cycle. The test results are illustrated in Figure 3c,d. Remarkably, the average particle hardness of SS‐NCA cathode particles exceeds that of RS‐NCA under diverse charging and cycling conditions. However, the drop of mechanical strength between electrochemical active state and pristine fresh state may be attributed to electrolyte impregnation, and the subsequent strength reduction can be ascribed to the accumulation of mechanical strain caused by repeated Li^+^ insertion and extraction.^[^ ^37^ ^]^ Whereas, at the charge level of 3.8 V, which is approximately at 50% state of charge (SoC) for the cathode, both NCA particles experience a rapid reduction in mechanical strength due to the *c*/*a* anisotropic lattice evolution present at this charging state,^[^ ^11^, ^19^ ^]^ which leads to an unstable cellular lattice structure and consequently decreases the mechanical stability of the charged cathode particles.^[^ ^37^, ^38^ ^]^ However, at all events, SS‐NCA exhibit well‐enhanced particle mechanical strength compared to that of RS‐NCA at both 3.8 and 4.3 V, as well as after five cycles at 2.7 V. Hence, the rational arrangement of grains and boundaries in the SS‐NCA cathode particle have reinforced its mechanical strength, which mitigated the particle cracking and thus reduced side‐reactions with electrolytes during cycling. This is beneficial in preventing detrimental phase transitions of the cathode under high de‐lithiation states and achieving superior electrochemical behaviors of the NCA cathode.

In situ X‐ray diffraction (in situ XRD) testing and Rietveld‐refined lattice parameters were conducted during the charging/discharging process of SS‐NCA and RS‐NCA cathodes to validate the impact of particle strength enhancement on suppressing irreversible structural distortions (see Figure 3e–g; Figure S10 and Table S3, Supporting Information). Overall, the SS‐NCA cathode demonstrates a refrained structural deterioration as compared with RS‐NCA cathode at the fully‐charged state (Figure 3e,f). The (101) peak shifts toward a higher angle during delithiation process due to the transition metal (TM) oxidation, leading to a shrink of the TM‐O bond and a decrease of the lattice parameter *a* (Figure 3g). The (003) peak displays a lower angle shift in low‐voltage regions during Li^+^ extraction, which is attributed to the increased repulsion between adjacent oxygen planes during charging. This results in an increase of the lattice parameter *c* (Figure 3g) and the corresponding shift of the (003) peak. While starting from 3.7 V, the (003) peak shifts back to higher angle, indicating a contraction of the *c* parameter at high SoC, accompanied by the unit‐cell volume decrease. The mechanism accounting for such a contraction during deep charge can be imputed to the phase transformations of “H1→H2→H3” processes and the regional “lattice collapse” at high SoC of a layered cathode.^[^ ^39^, ^40^, ^41^ ^]^ Based on the Rietveld refinement results, it can be observed that during the charging process, the SS‐NCA cathode exhibits a smaller shift of the (003) peak (*∆*2*θ* = 0.61^o^) compared to the RS‐NCA cathode (*∆*2*θ* = 0.70^o^). Additionally, the contraction rate of both lattice parameter *c* (−2.42% for SS‐NCA and −2.59% for RS‐NCA) and unit‐cell volume for SS‐NCA cathode (−6.50% for SS‐NCA and −6.66% for RS‐NCA) is much lower than that of RS‐NCA cathode. These results suggest that the particle strength enhanced SS‐NCA provides greater benefits than RS‐NCA in preventing adverse structural changes of the cathode by avoiding excessive intrusion of electrolyte.^[^ ^40^, ^41^ ^]^ While, the variation patterns of lattice parameters during discharge are correspondingly reversed to those of the aforementioned charging electrochemical processes, and demonstrating a superior electrochemical reversibility of the SS‐NCA cathode as compared to RS‐NCA cathode.

Furthermore, the high‐precision leakage current tester was used for SS‐NCA and RS‐NCA to study the parasitic reactions between the cathode particles and electrolyte. In the leakage current system, the coin cells of SS‐NCA and RS‐NCA cathodes were conditioned at 25 °C in a thermostatic chamber, and a high‐precision source meter (Keithley 2401) was utilized to charge the cells up to a high potential of 4.5 V and maintain this potential for 45 h. The schematic inset depicted in Figure 3h illustrates the fundamental principle of the leakage current measurement. The state of charge (SoC) or the lithium concentration in the working electrode will reach an equilibrium state after the constant‐voltage charging and holding by the source meter. During this process, the electron obtained from the environment through solvent oxidation will be electrochemically monitored by the external circuit (Keithley 2401). The measured leakage current is practically proportional to the reaction rate of side reactions between the working electrode and electrolyte. Therefore, the static leakage current can serve as a quantitative indicator of the reaction rate of side reactions.^[^ ^41^, ^42^ ^]^ Figure 3h displays the current relaxation curves collected from the SS‐NCA and RS‐NCA cathodes, where an exponential decay function was used to extract the static side reaction current of *y*_0_ in *y* = A^*^exp(−x/t) + *y*_0_. The resulting values for the static leakage currents (y_0_) of SS‐NCA and RS‐NCA cathodes were found to be 1.68 and 2.29 µA mg^−1^, respectively. These findings suggest that parasitic reactions between SS‐NCA cathode particles and electrolyte have been mitigated through the reinforcement modification of particle internal structures as compared to RS‐NCA.

The changes in the kinetics of Li‐ion transport due to the proportion difference of SAMGBs constructed in cathode particles were investigated by using galvanostatic intermittent titration technique (GITT) measurements.^[^ ^43^ ^]^ The GITT curves of the SS‐NCA and RS‐NCA cathodes were gathered during charging/discharging with a pulsed current at C/3 for 10 min followed by 2 h rest to reach the equilibrium state (Figure 3i). The Li^+^ diffusion coefficients (*D*_Li+_) of SS‐NCA and RS‐NCA cathodes, which were calculated from the GITT curves,^[^ ^43^, ^44^ ^]^ were plotted on a logarithmic scale as shown in Figure 3j. At lower delithiation states, the *D*_Li+_ values of SS‐NCA and RS‐NCA cathodes are similar. Whereas, at higher delithiation states, the *D*_Li+_ values of the SS‐NCA cathode are notably larger than those of the RS‐NCA cathode during both charging and discharging processes. This phenomenon suggests that the kinetics of Li‐ion transport in SS‐NCA cathode is improved, especially at higher SoCs. This can be explained by that the increasing construction of SAMGBs within SS‐NCA particles is conducive to promoting facile inter‐grain Li transfers,^[^ ^34^, ^35^ ^]^ and the enhanced mechanical strength of particles with improved layered structure stability at high SoC are beneficial to repress adverse phase transitions caused by electrolyte corrosion,^[^ ^37^ ^]^ as confirmed by the in situ XRD and leakage current measurements.

### 2.3 Electrochemical Performance and Cathode Structural Robustness Characterization

**Figure 4:**
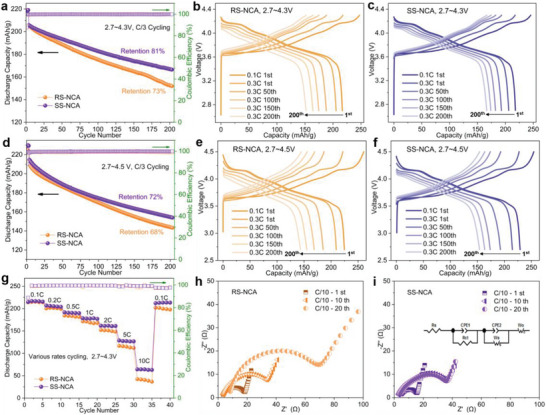
Electrochemical performances of RS‐NCA and SS‐NCA cathodes. Cycling performance of the RS‐NCA and SS‐NCA cathodes in the voltage range a–c) 2.7–4.3 V and d–f) 2.7–4.5 V, with half‐cell cycled at C/3 following three formation cycles at C/10. a) Specific capacity retention after 200 cycles. b,c) Evolution of the charge/discharge curves of RS‐NCA (b) and SS‐NCA (c) cathodes between 2.7 – 4.3 V. d) Specific capacity as a function of cycle numbers when cycled between 2.7–4.5 V. e,f) Evolution of the charge/discharge curves of RS‐NCA (e) and SS‐NCA (f) cathodes cycling between 2.7–4.5 V. g) High‐rate performance of the cathodes at various rates (0.1, 0.2, 0.5, 1, 2, 5, and 10 C). h,i) Impedance spectra evolution of the RS‐NCA electrode (h) and the SS‐NCA electrode (i) at the 1st, 10th, and 20th cycles. Insert in (i) is the equivalent circuit used to fit the impedance data.

The electrochemical performances of the SS‐NCA and RS‐NCA cathodes (LiNi_0.90_Co_0.08_Al_0.02_O_2_) were evaluated in 2032 coin‐type half cells using Li metal as the anode, within the voltage range of 2.7–4.3 V (**Figure** **4**a–c) and 2.7–4.5 V (Figure 4d–f), respectively. As shown in Figure 4a, the SS‐NCA cathode exhibited superior cycle stability compared to the RS‐NCA cathode. It delivered a high initial specific capacity of 245.3 mAh g^−1^ on charge and 217.9 mAh g^−1^ on discharge (88.8% initial Coulombic efficiency, ICE) at C/10 rate (1 C = 200 mA g^−1^) between 2.7–4.3 V. After 200 cycles at C/3 rate, a capacity retention of 81% was achieved. By contrast, the RS‐NCA cathode exhibited a deteriorated capacity retention with only 73% remaining after 200 cycles at C/3 rate. While the initial specific capacities of RS‐NCA cathode were 247.3 mAh g^−1^ on charge and 216.7 mAh g^−1^ on discharge (87.6% ICE) at C/10, both of the initial discharge capacity and ICE were lower than those of the SS‐NCA cathode. Moreover, as revealed by the evolution of the charge/discharge curves depicted in Figure 4,c, RS‐NCA cathode has dramatically accelerated voltage decey (Figure S11, Supporting Information) and severe capacity loss during cycling in contrast to the SS‐NCA cathode. These observations indicate that tailoring beneficial alignment of grains and reinforcing particle strength in the SS‐NCA cathode has a vital effect on the electrochemical performance enhancement. Furthermore, when cycling at an upper voltage range of 2.7–4.5 V at C/3 rate, as displayed in Figure 4d–f, the SS‐NCA cathode still demonstrated a higher capacity retention (72%) than that of the RS‐NCA cathode (68%) after 200 cycles. Noticeably, between the voltage range of 2.7–4.5 V, the SS‐NCA cathode exhibited significantly higher ICE (90.0%) and higher initial discharge capacity (230.1 mAh g^−1^) in comparison to those of the RS‐NCA cathode (ICE 86.2%, initial discharge capacity 224.6 mAh g^−1^). The voltage decay (Figure S12, Supporting Information) and capacity loss during cycling were still mitigated in SS‐NCA cathode. The improved upper voltage electrochemical performance of SS‐NCA cathode is consistent with the high‐precision leakage current results (Figure 3h), which is credited to that grain structure modifications and particle strength intensifications have effectively suppressed the parasitic reactions between SS‐NCA cathode particles and electrolyte, resulting in stable and reversible electrochemical reactions at higher voltages.^[^ ^10^, ^42^, ^44^ ^]^ In addition, the SS‐NCA cathode exhibited an outstanding high‐rate capability at various rates of 0.5, 1, 2, 5, 10 C rate as compared to the RS‐NCA cathode (see Figure 4g; Figure S13, Supporting Information). As shown in Figure 4g, after 40 cycles at various rates cycling (0.1, 0.2, 0.5, 1, 2, 5, 10, 0.1 C rate, respectively), the SS‐NCA cathode can still deliver a large discharge capacity of 213.6 mAh g^−1^ at C/10 with a high capacity retention of 99.4%, suggesting the good tolerance of various abuse uses to maintain good cycle stability. On the contrary, the discharge capacities at various rates are much lower, and the capacity retention is only 91.2% after 40 cycles at various rates for the RS‐NCA cathode. Figure 4h,i presented electrochemical impedance spectra (EIS) evolution of the SS‐NCA and RS‐NCA cathodes tested at completely discharged state in different cycles at C/10 rate. Both the Nyquist plots of the SS‐NCA and RS‐NCA cathodes in the high‐frequency region showed a semicircle profile, which were assigned to the surface charge‐transfer process in the cathodes.^[^ ^45^ ^]^ As calculated from the EIS data, the surface charge‐transfer resistances (R_ct_) of the SS‐NCA cathode in different cycles were 16.1 Ω, 34.7 Ω, 36.3 Ω at the 1st, 10th, and 20th cycles, respectively; While for the RS‐NCA cathode, they were found to be 19.6, 35.0, 70.8 Ω, respectively. This suggests that the charge‐transfer resistance at the cathode/electrolyte interface increased more rapidly for the RS‐NCA cathode than for the SS‐NCA cathode, implying inferior ionic/electronic conductivity resulting from particle cracking and interface side‐reactions with electrolyte during cycling of the RS‐NCA cathode without particle strength reinforcement modification with surface densely‐packed random‐aligned grains.^[^ ^37^, ^45^, ^46^ ^]^

**Figure 5:**
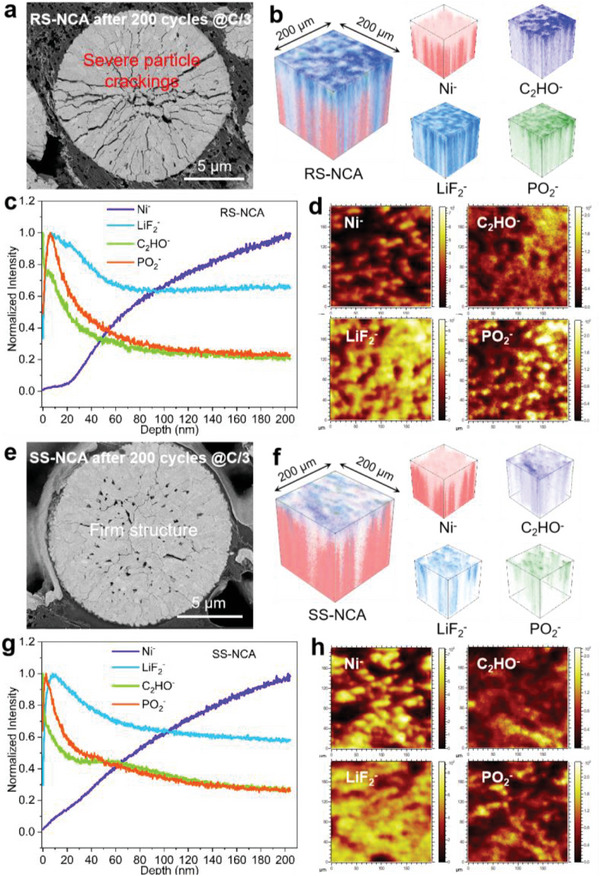
Structure robustness and parasitic evolution of RS‐NCA and SS‐NCA cathodes after 200 cycles between 2.7–4.5 V at C/3. a,e) SEM images of cross‐sectional cathode particles of RS‐NCA (a) and SS‐NCA (e) after 200 cycles. b,f) 3D ToF‐SIMS images visualization of secondary‐ion fragments for the RS‐NCA (b) and SS‐NCA (f) after 200 cycles. c,d) ToF‐SIMS depth profiling and chemical maps of Ni^−^, C_2_HO^−^, LiF_2_^−^, and PO_2_^−^ on the RS‐NCA cathode after 200 cycles. g,h) ToF‐SIMS depth profiling and chemical maps of Ni^−^, C_2_HO^−^, LiF_2_^−^, and PO_2_^−^ on the SS‐NCA cathode after 200 cycles.

Besides, the mechanical structure robustness and parasitic evolution of the SS‐NCA and RS‐NCA cathodes after 200 cycles were revealed by employing SEM and time‐of‐flight secondary‐ion mass spectrometry (ToF‐SIMS) measurements (**Figure** **5**). The cross‐sectional SEM images of the RS‐NCA and SS‐NCA cathode particles after 200 cycles are displayed in Figure 5a,e. Remarkably, the SS‐NCA cathode particle still maintained the intact spherical particle structure after long‐term cycling as shown in Figure 5e. In contrast, significant microcracking occurred within the RS‐NCA cathode (Figure 5a), extending from the interior to the surface of particle due to repeated cell volume variations during charging and discharging.^[^ ^25^, ^27^ ^]^ The drastic microcracks propagated in the RS‐NCA cathode particle not only electrically divorce the ion‐conducting pathways between inter‐grains, but also create channels for electrolyte penetration to the particle interior, which leads to an increase in interfacial resistance by growing of hindered boundaries and serious parasitic structural degradation of the cathode caused by electrolyte corrosion.^[^ ^24^, ^25^, ^26^, ^27^, ^39^, ^40^, ^41^, ^46^ ^]^ In fact, the grain surface of RS‐NCA cathode after 200 cycles suffered from much severe phase transitions into rock‐salt and disordered structures in comparison to that of the SS‐NCA cathode, as shown in the high‐resolution TEM (HRTEM) images (see Figure S14, Supporting Information). Furthermore, to gain in‐depth understanding of the depraved parasitic evolution of SS‐NCA and RS‐NCA cathodes imputed by particle cracking and electrolyte attack, ToF‐SIMS were performed on the two cathodes after 200 cycles to investigate the structure and chemistry of the passivated cathode‐electrolyte interface (CEI) layers.^[^ ^47^ ^]^ The secondary‐ion fragments of C_2_HO^−^, LiF_2_^−^, and PO_2_^−^ were detected to signify the deteriorative side‐reaction products of electrolyte solvent and salt on the cathode surface.^[^ ^48^ ^]^ The Ni^−^ fragments were employed to indicate the bulk NCA cathode particle.^[^ ^48^ ^]^ Figure 5c,g show the normalized depth profiles of the detected fragments for the cycled SS‐NCA (Figure 5g) and RS‐NCA (Figure 5c) cathodes over a depth of 200 nm, corresponding to a sputter time of 1200 s at a sputter rate of 0.17 nm s.^–1[^ ^47^ ^]^ Apparently, with the increase in detecting depth (see Figure 5c,g), the intensity signals of C_2_HO^−^, LiF_2_^−^, and PO_2_^−^ fragments exhibit a profile of increasing at first and then decreasing to level off, while the Ni^−^ fragment signals display a crescent growing profile, confirming the presence of a parasitical CEI layer originated from electrolyte side‐reactions on the bulk surface of the SS‐NCA and RS‐NCA cathode particles.^[^ ^24^, ^25^, ^26^, ^27^, ^47^, ^48^ ^]^ As demonstrated in the ToF‐SIMS chemical maps (see Figure 5d,h), compared with the cycled SS‐NCA cathode, the cycled RS‐NCA cathode show much weaker signals of Ni^−^ species and stronger signals of C_2_HO^−^, LiF_2_^−^ and PO_2_^−^ species, suggesting the surface passivated CEI layers overlayed on the RS‐NCA cathode are much thicker than that on the SS‐NCA cathode,^[^ ^39^, ^40^, ^41^, ^47^, ^48^ ^]^ which are also verified by the 3D‐rendering ToF‐SIMS visualization images as depicted in Figure 5b,f, respectively. In conclusion, the poor mechanical performance of the RS‐NCA cathode results in severe particle cracking during cycling, which promotes pernicious parasitic‐reactions between the RS‐NCA cathode particles and electrolyte. This triggers the formation of an electrochemically‐inert rock‐salt structured NiO phase with high impedance on the cracking surface of the cathode,^[^ ^24^, ^25^, ^26^, ^27^, ^39^, ^41^ ^]^ leading to the speedy increasing in resistance and the rapid decline in capacity and voltage during cycling for the RS‐NCA cathode. However, benefiting from the enhanced particle mechanical stability, the SS‐NCA cathode can effectively suppress the above‐mentioned electrochemical failure processes and achieve superior cycle stability.^[^ ^37^, ^38^ ^]^

**Figure 6:**
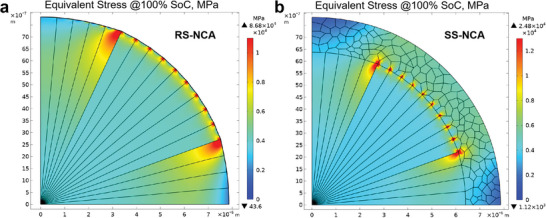
Finite element simulation of the equivalent stress distribution in the cathode particles of a) RS‐NCA and b) SS‐NCA at fully‐charged state.

Furthermore, finite element modeling (FEM) simulation was conducted to study the stress distributions within the cathode particles of the RS‐NCA and SS‐NCA cathodes at various states of charge from 0% to 100% SoCs (see Figure S15 and Movies S1 and S2, Supporting Information). Due to the extensive release of Li^+^ ions and the resulting cell volume change with large anisotropic strains, the build‐up of inner stress within the cathode particle occurs distinctly at 100% SoC as demonstrated by our FEM simulation results (**Figure** **6**). As shown in Figure 6a, without the primary‐grain‐alignment modification with a shield of dense random‐oriented grains, the stress distribution in the RS‐NCA cathode particle mainly concentrates on the surface of the particle that growing out from inner stress cumulations with along the GBs of radially arrayed grains, making it vulnerable to particle splitting and electrolyte assaulting into the cathode particles.^[^ ^28^, ^49^ ^]^ Remarkably, the stress concentration in the SS‐NCA cathode particle dominatingly located at the interface of the radially arrayed grains and the rim‐coated random‐oriented grains, which is trapped within the particle interior and not extend to the particle surface as exhibited in Figure 6b. The abundant inter‐grain boundaries formed by densely packed small‐sized random‐oriented grains shielded on the SS‐NCA particle can serve as barriers against the propagation of inner microcracks delivering to the surface. Therefore, the unique microstructure of the SS‐NCA cathode, with effective mediation of microstrains within the particle, is crucial in inhibiting particle cracking in the deeply charged state, which makes great sense to improve the cycling stabilities of the cathode by avoiding further electrolyte attacks.^[^ ^24^, ^25^, ^26^, ^27^, ^39^, ^41^ ^]^

### 2.4 Oxygen Evolution Analysis

**Figure 7:**
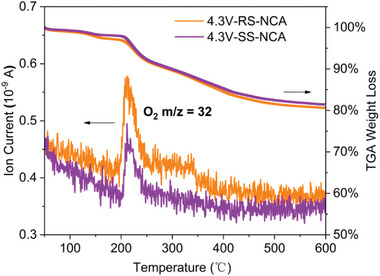
Measurements of oxygen release for charged cathodes by thermogravimetric analysis combined with mass spectrometry (TGA‐MS). Thermogravimetric (TG) profiles and corresponding oxygen release of the RS‐NCA and SS‐NCA cathodes charged at 4.3 V.

Generally, the electrolyte decomposition on the parasitic cathode/electrolyte interface and the induced oxygen evolution from the newly exposed surface on oxide microcracks can potentially trigger thermal runaway of batteries.^[^ ^10^, ^50^, ^51^ ^]^ Hence, strengthening the surface oxygen, especially at highly delithiated state, is a key factor for ensuring the structural and thermal stability of Ni‐rich oxide cathodes.^[^ ^10^, ^11^, ^20^ ^]^ In this context, the thermogravimetric analysis combined with mass spectrometry (TGA‐MS) was conducted to investigate the lattice oxygen stability and thermal behavior of the SS‐NCA and RS‐NCA cathodes charged at 4.3 V. As displayed in **Figure** **7**, the thermogravimetric (TG) profiles of the charged cathode powders revealed that the charged RS‐NCA cathode started to experience weight loss at ≈160°C due to oxygen escape,^[^ ^52^, ^53^ ^]^ which occurs apparently earlier than that of the charged SS‐NCA cathode (>180 °C). Accompanied with the TG weight loss, the detected oxygen liberation in the RS‐NCA cathode originating from the thermal decomposition reaction of charged Ni‐rich oxide is much more serious than that in the SS‐NCA cathode, suggesting the improved structural and thermal stability of the charged SS‐NCA cathode by grain engineering that could avoid newly exposed surface oxygen‐release boundaries from microcracks at high SoC.^[^ ^10^, ^52^, ^53^ ^]^ Moreover, the DSC‐MS measurements (as depicted in Figure S16, Supporting Information) have also confirmed the enhanced thermal stability of the SS‐NCA cathode with lower heat release and less oxygen liberation than those from the RS‐NCA cathode. In consequence, by proper engineering the grain orientation and integrating assembly within polycrystalline particles, it is highly enlightening in promoting the thermal safety performance of the Ni‐rich oxides cathode.

## 3 Conclusion

In summary, in order to stabilize the bulk structure and achieve the desired electrochemical and safety performance of Ni‐rich layered oxide cathode, we propose a facile strategy involving reasonable design of internal crystallographic orientation of microdomains within polycrystalline particle with well‐enhanced mechanical strength and Li‐ions transfer kinetics, without resorting to any taxing processes of foreign elements doping and surface heterophase coating. Uniquely, the inner‐core section of the strategically designed surface‐shielded NCA is composed of tightly radially‐oriented grains, while the outer‐rim section consists of densely packed randomly‐oriented grains, as can be clearly demonstrated by the cross‐sectional SEM and EBSD technique. Importantly, the alignment of radially‐oriented grains with preferential crystalline a–b planes along Li slabs to point outward from the particle center, as well as the construction of significant SAMGBs with easy inter‐grain Li transfers, promotes efficient lithium‐ion transport kinetics in the SS‐NCA cathode during cycling. Notably, the synergetic orientation arrangements of primary grains have played a critical role in intensifying the mechanical strength of cathode particles, through which the surface‐shielded densely packed small‐size random‐oriented grains with abundant inter‐grain boundaries serve as barriers to interrupt the inner microcracks propagating to the surface induced by intrinsic stress accumulation between radial‐aligned grains, as systematically confirmed by the single‐particle micro‐compression tests, finite element modeling (FEM) simulations, and cross‐sectional SEM of the cathodes after 200 cycles. By utilizing in situ XRD during charging/discharging, high‐precision leakage current measurements, and ToF‐SIMS spectrometry, the enhancement in mechanical strength of cathode particles are verified highly at works to restrain irreversible structural degradation of the cathode and mitigate passivating side‐reactions with electrolyte by avoiding newly exposed parasitic surface on oxide microcracks. Taking advantages of these established favorable factors, the SS‐NCA cathode exhibits markedly improved cycling stability and rate capability, and restrained resistances growth in comparison to the conventional RS‐NCA cathode, as demonstrated by the electrochemical characterizations, correspondingly. In addition, the reduced electrolyte decomposition on the parasitic cathode/electrolyte interface and the suppressed particle splitting with less exposure of oxygen‐releasing boundaries at high de‐lithiation state, have enhanced the thermal stability of the charged SS‐NCA cathode by strengthening the lattice oxygen stabilities as affirmed by the TGA‐MS and DSC‐MS measurements. This study will trigger interest in both intrinsic understanding and practical optimization by tailoring the grain arrangement and boundary configuration in polycrystalline cathodes for exceptional electrochemical and safety performances of lithium‐ion batteries.

## 4 Experimental Section

### Materials and Synthesis

The preparation of the transition metal hydroxide Ni_0.92_Co_0.08_(OH)_2_ precursors for the SS‐NCA and RS‐NCA were supported by CNGR advanced material Co., Ltd, with impurity elements <500 ppm, respectively. As for the synthesis of the SS‐NCA, the precursor was dried at 110°C for 10 h in a vacuum oven, followed by thorough mixing with LiOH and 2% mol Al(OH)_3_, and then calcinating under constant oxygen flow at 2.0 L min^−1^ in a tube furnace. An additional 5% of LiOH was used to compensate for potential loss of lithium at high temperatures. The sample was heated from 25 to 550 °C and held for 2 h, and further heated to 710 °C and held for 12 h. The heating rate was set at 2 °C min^−1^. Then, the furnace was cooled to 25 °C with cooling rate of 1°C min^−1^ under constant oxygen flow to obtain the SS‐NCA powders. The RS‐NCA powders were also obtained by following the identical synthesis procedures and conditions as above mentioned.

### Electrochemical Measurements

The composite cathodes slurry was prepared using 90% active material, 5% polyvinylidene fluoride (PVDF), and 5% acetylene carbon black in *N*‐methyl‐2‐pyrrolidone (NMP). The obtained slurry was coated on the current collector (Al foil, 16 µm in thickness) and dried in a vacuum oven at 110 °C for 12 h. The cathodes were punched into small discs with area of 78.5 mm^2^, and the mass loading of the cathodes were precisely controlled within 6.45–6.55 mg cm^−2^. In the flowing stamping process, the compaction density of the cathodes were precisely controlled within 3.15–3.20 g cm^−3^. In a glovebox with high‐purity Ar, CR2032‐type coin cells were assembled to characterize the electrochemical performance of the cathodes. The electrolyte was composed of LiPF_6_ (1 m) in ethylene carbonate (EC) and ethyl methyl carbonate (EMC) mixture solvents (3:7 v/v). Li metal was adopted as the anode. The Celgard 2500 polypropylene (PP) porous membrane was served as the separator. The electrochemical testing of coin cells was conducted on a LAND‐CT2001A battery tester under various charge/discharge rates (1 C = 200 mAh g^−1^) in the cutoff voltage range of 2.7–4.3 and 2.7–4.5 V. The electrochemical impedance spectra (EIS) was performed on an electrochemical workstation (Zahner IM6e) with 5 mV amplitude of perturbation in the frequency range of 10 kHz to 10 MHz.

### Materials Characterization

The X‐ray diffraction patterns of the samples were obtained by using an X‐ray diffractometer (Bruker Advance D8) with Cu Kα radiation (*λ* = 1.5418 Å) in the scan range (2*θ*) of 5–85°. The Rietveld refinement of crystal structure parameters was conducted using the General Structure Analysis System (GSAS) program. For in situ XRD test during charging and discharging, a specially designed electrochemical‐reaction cell equipped with an Al window for X‐ray penetration was assembled. The in situ XRD patterns were collected every 30 min within 2*θ* range from 5° to 85° and voltage range of 2.7‐4.3 V (vs Li^+^/Li) under current rate of 0.1 C. The morphology and microstructure of all the samples were characterized by using a scanning electron microscope (SEM, JEOL‐JSM7800F). The mechanical durability of the cathode particles (pristine particles and electrochemically‐processed particles) was analyzed using a micro‐compression tester (MCT‐210, Shimadzu). As for the obtaining of the electrochemically‐processed particles, the cycled cells were disassembled in an Ar‐filled glove box and the collected cathodes were thoroughly washed with dimethyl carbonate (DMC) solvent to eliminate the residual salts. After further washed with acetone and dried at 100 °C for 10 h in a vacuum oven, the electrochemically‐processed cathode particles were peeled off from the cathodes. ToF‐SIMS (ION‐ToF GmbH, Germany) were utilized with a pulsed 30 keV Bi^3+^ as the primary ion beam. The beam current was 1.04 pA at a repeating frequency of 10 kHz to scan on a square area of 200 × 200 µm^2^ of the cathode surface. The thermal stability of the cathode materials were measured by both TG‐MS (Thermogravimetry‐Mass Spectrometry, TG: STA449F5 from NETZSCH Ltd., MS: T200 from TILON GRP Technology Ltd.,) and DSC‐MS (Differential Scanning Calorimetry‐Mass, DSC: STA449F5 from NETZSCH Ltd., MS: QMS403D from NETZSCH Ltd.,). The heating rates in TG‐MS and DSC‐MS measurements for the tested samples were kept the same at 10°C min^−1^.

### Finite Element Modeling

To simulate Li diffusion and stress evolution in the RS‐NCA and SS‐NCA particles, a feasible 2D plane electrochemical–mechanical strain model was built. In the model, the NCA particle was depicted as a circular domain consisting of numerous grains. The *a*‐axis of radially oriented grains were arranged in a radial alignment, while the *a*‐axis of randomly oriented grains were arranged in a packed random alignment. Both Li diffusion and induced strain demonstrated high anisotropy. The lattice defects of anti‐sites were pre‐set in the model with a concentration of 1%. The Li‐ion diffusivities along *a*‐axis and *c*‐axis were set as 7 × 10^−15^ and 7 × 10^−16^ m^−1^s^2^, respectively, according to those of NCA materials. The Li diffusion‐induced strains at 100% SoC in the *a*‐axis were respectively set as −2.12% for the RS‐NCA and −2.16% for the SS‐NCA, while those in the *c*‐axis were respectively set as −2.59% for the RS‐NCA and −2.42% for the SS‐NCA, according to the Rietveld refinement results from the in situ XRD tests (Figure 3g). The surface of the NCA particle was imposed by an outflux of Li, which was calculated based on the current rate of C/5. The commercial software COMSOL Multiphysics V5.3 was used to solve the governing equations for the deformation kinematics and the diffusion kinetics of the cathode particle.

## Conflict of Interest

The authors declare no conflict of interest.

## Supporting information

Supporting Information

Supplemental Movie S1

Supplemental Movie S2

Supplemental Movie S3

Supplemental Movie S4

## Data Availability

The data that support the findings of this study are available from the corresponding author upon reasonable request.
