# Supplementary material for: Enabling Electrochemical–Mechanical Robustness of Ultra‐High Ni Cathode via Self‐Supported Primary‐Grain‐Alignment Strategy
Source: Adv Sci (Weinh). 2023 Oct 26;10(36):2306347. doi: 10.1002/advs.202306347 (PMC10754075; doi:10.1002/advs.202306347)
Supplement: Supplementary file 1 — Supporting Information [file ADVS-10-2306347-s004.pdf]

ADVANCED SCIENCE
Open Access

Supporting Information

for *Adv. Sci.*, DOI 10.1002/advs.202306347

Enabling Electrochemical–Mechanical Robustness of Ultra-High Ni Cathode via Self-Supported Primary-Grain-Alignment Strategy

Yu-Kun Hou, Chenxi Li, Dongsheng Ren, Feixiong He, Kaijun Zhuang, Shuo Yin, Guohe Yuan, Yiqiao Wang, Yi Guo, Saiyue Liu, Peng Sun, Zhihua Zhang, Tiening Tan, Gaolong Zhu, Languang Lu, Xiang Liu* and Minggao Ouyang**

Supporting Information

Enabling Electrochemical-Mechanical Robustness of Ultra-High Ni Cathode via Self-supported Primary-grain-alignment Strategy

Yu-Kun Hou[1,3], Chenxi Li[2], Dongsheng Ren[1]*, Feixiong He[3], Kaijun Zhuang[1,5], Shuo Yin[4], Guohe Yuan[4], Yiqiao Wang[4], Yi Guo[1], Saiyue Liu[1], Peng Sun[6], Zhihua Zhang[6], Tiening Tan[3], Gaolong Zhu[3], Languang Lu[1], Xiang Liu[2]*, and Minggao Ouyang[1]*

[1]School of Vehicle and Mobility, Tsinghua University, Beijing, 100084, China

[2]School of Materials Science and Engineering, Beihang University, Beijing, 100191, China

[3]Prof. Ouyang Minggao Academician Workstation, Sichuan new Energy Vehicle innovation Center Co., Ltd., Yibin, 644000, China

[4]CNGR advanced material Co., Ltd., Tongren, 554000, China

[5]School of Control and Computer Engineering, North China Electric Power University, Beijing, 102208, China

[6]Changzhou Institute of Advanced Manufacturing Technology, Changzhou, 213000, China

*Corresponding author:
Xiang Liu (xiangliu@buaa.edu.cn)
Dongsheng Ren (rends@mail.tsinghua.edu.cn)
Minggao Ouyang (ouymg@mail.tsinghua.edu.cn)

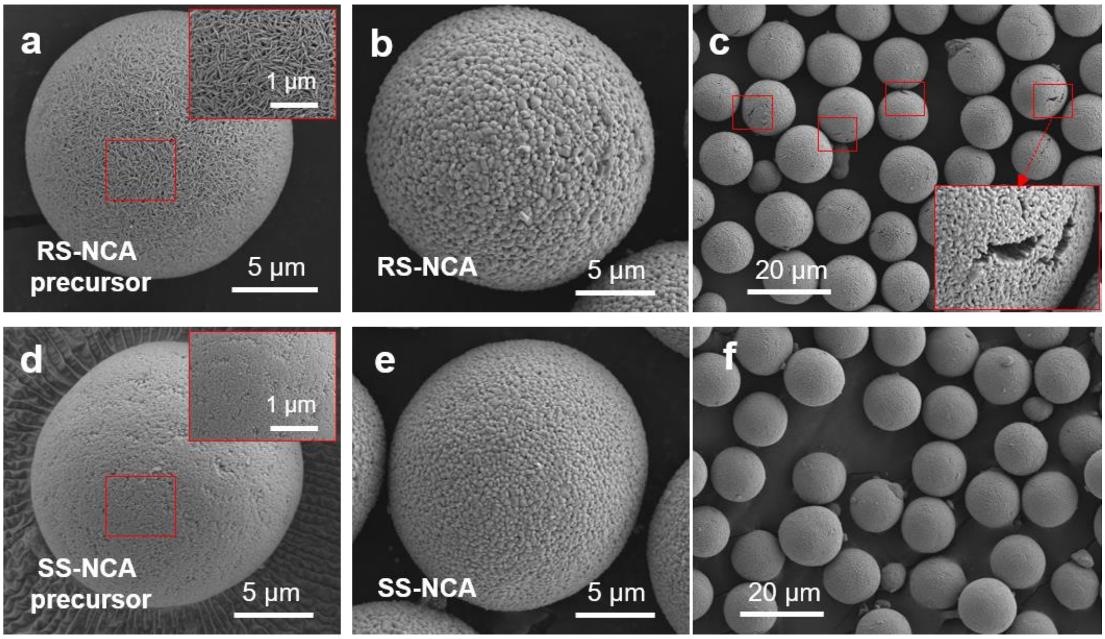

Figure S1. SEM image of precursor particle of RS-NCA (a) and SS-NCA (d). SEM image of cathode particles of RS-NCA (b,c) and SS-NCA (e,f).

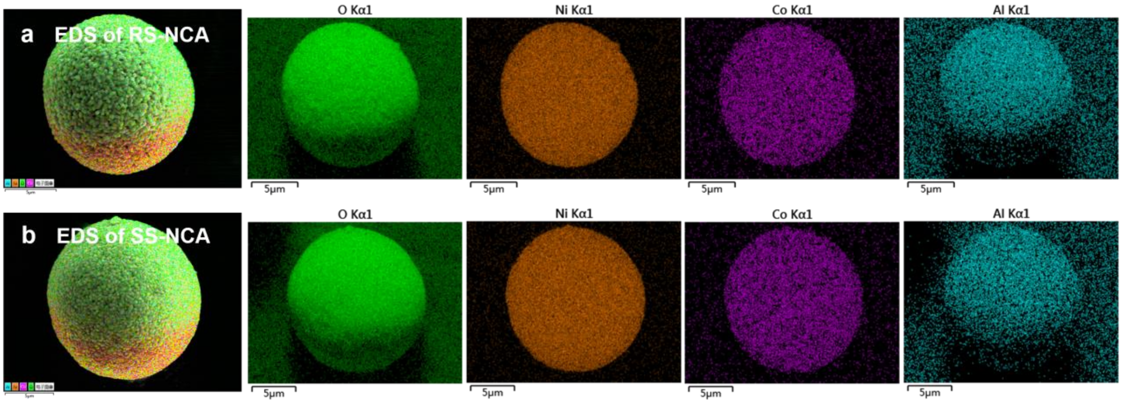

Figure S2. SEM energy dispersive spectroscopy (EDS) mappings of RS-NCA (a) and SS-NCA (b), showing uniform distributions of O, Ni, Co and Al elements on the as-prepared cathode particle of RS-NCA and SS-NCA, respectively.

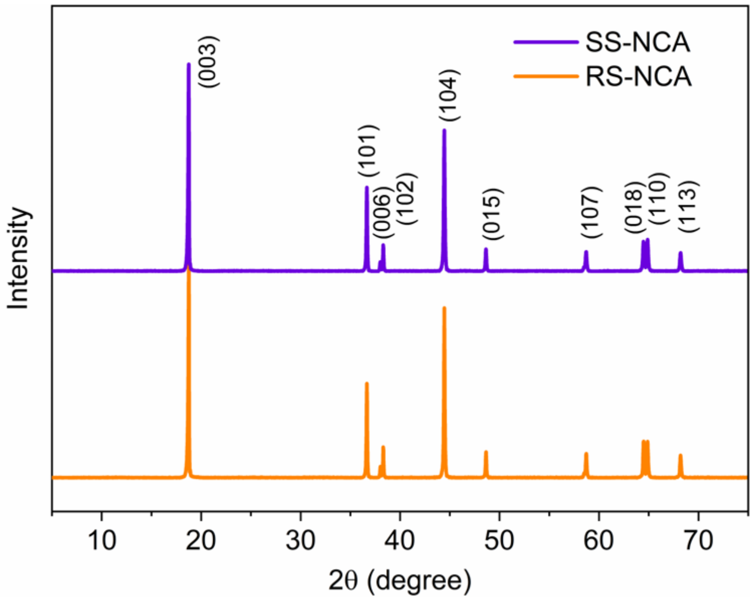

Figure S3. XRD patterns of the as-prepared RS-NCA and SS-NCA cathode materials.

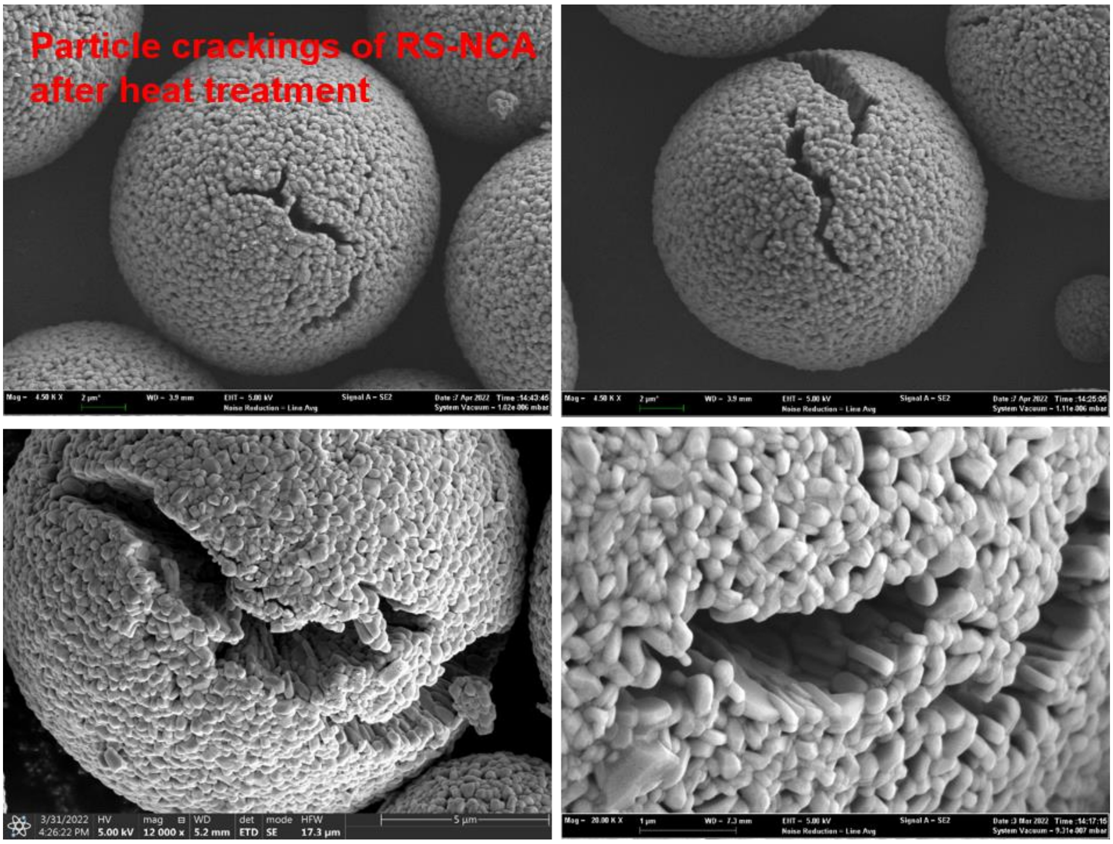

Figure S4. SEM image of cathode particle crackings of RS-NCA after heat treatment.

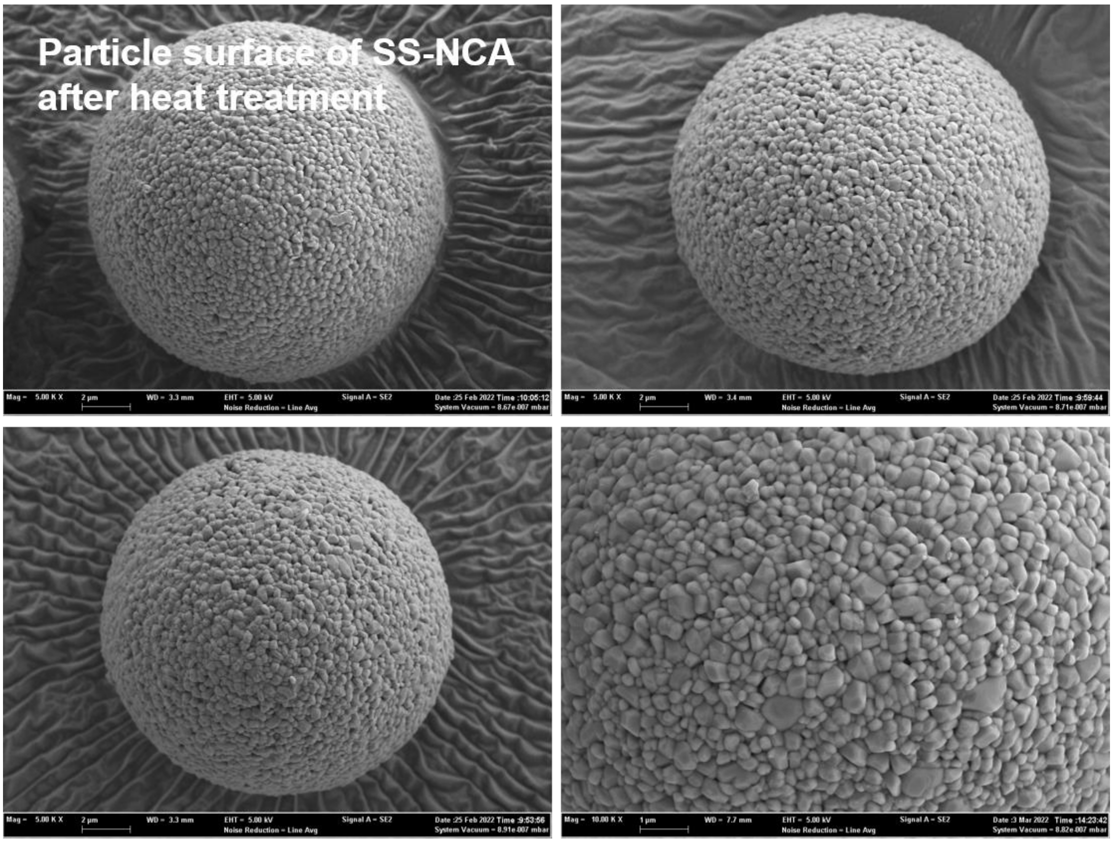

Figure S5. SEM image of cathode particle surface of SS-NCA after heat treatment.

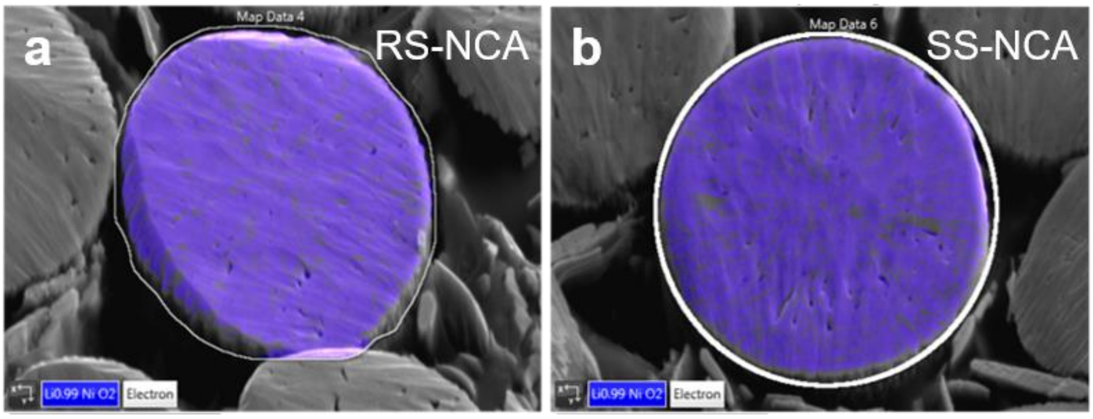

Figure S6. Backscatter electron diffraction (EBSD) testing areas for the RS-NCA (a) and SS-NCA (b) cathode particle.

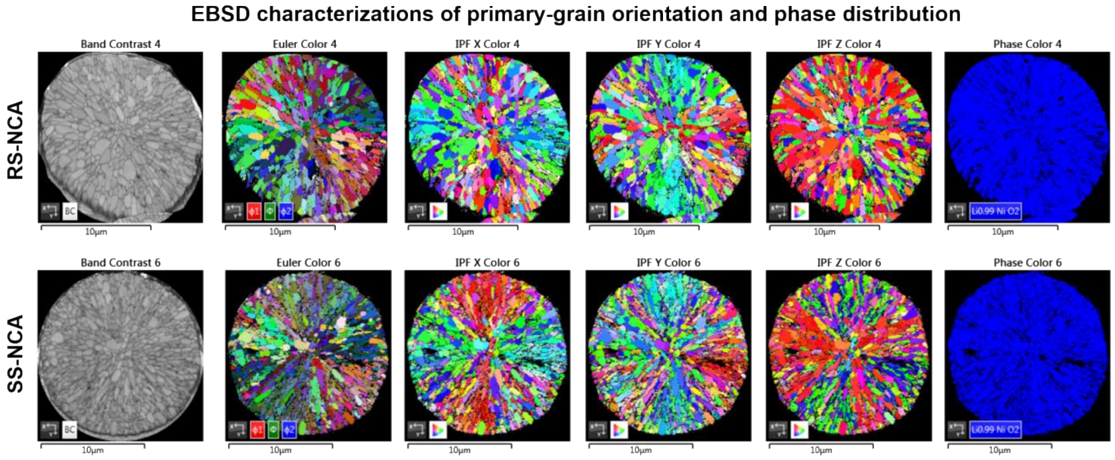

Figure S7. EBSD characterization of grain alignments, grain orientation distributions and phase distributions for the RS-NCA and SS-NCA.

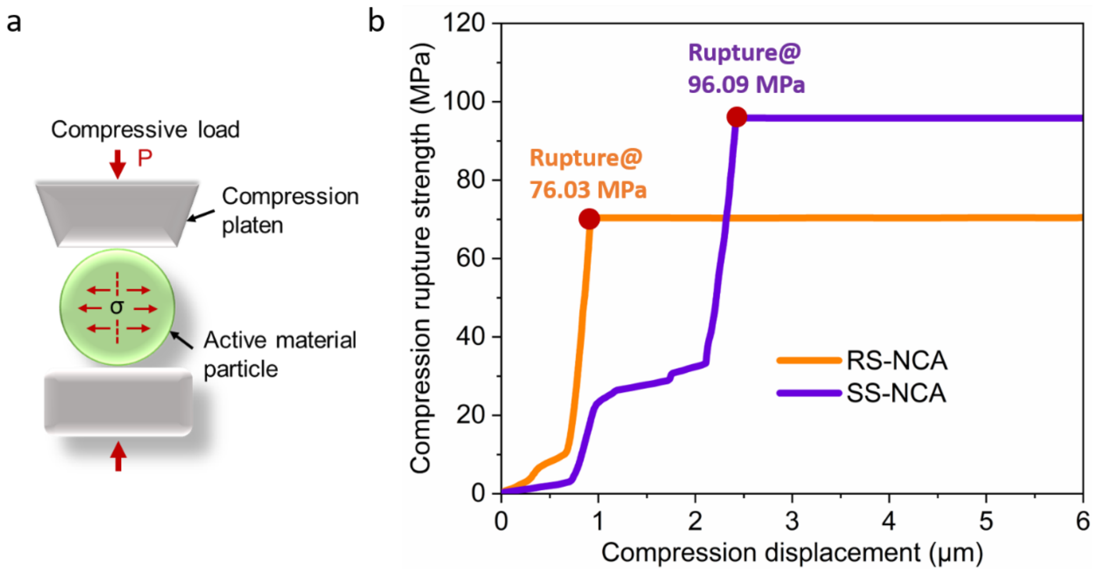

Figure S8. A typical compression force-displacement curves for single-particle of the RS-NCA and SS-NCA.

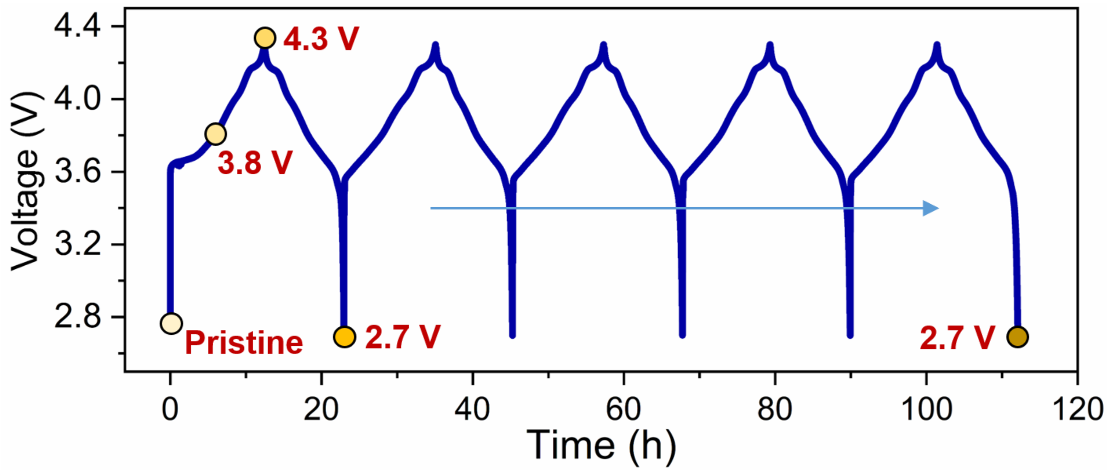

Figure S9. The different electrochemical states of cathode particles for the *ex-situ* single-particle micro-compression tests of SS-NCA and RS-NCA.

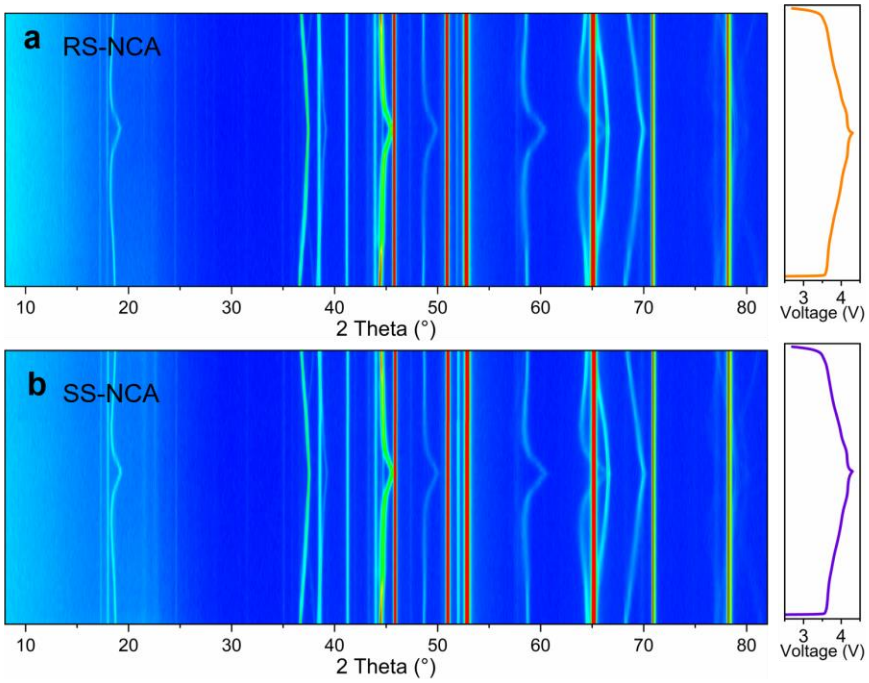

Figure S10. In situ XRD contour images of RS-NCA (a) and SS-NCA (b) cathodes during charge/discharge within 2.7 – 4.3 V at C/10.

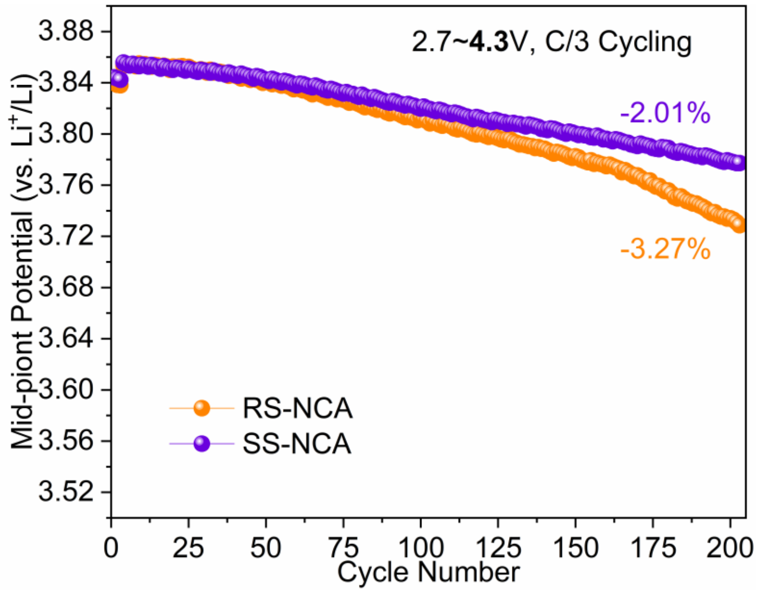

Figure S11. Evolution of discharge mid-point potential of RS-NCA and SS-NCA cathodes during C/3 cycling between 2.7 – 4.3 V.

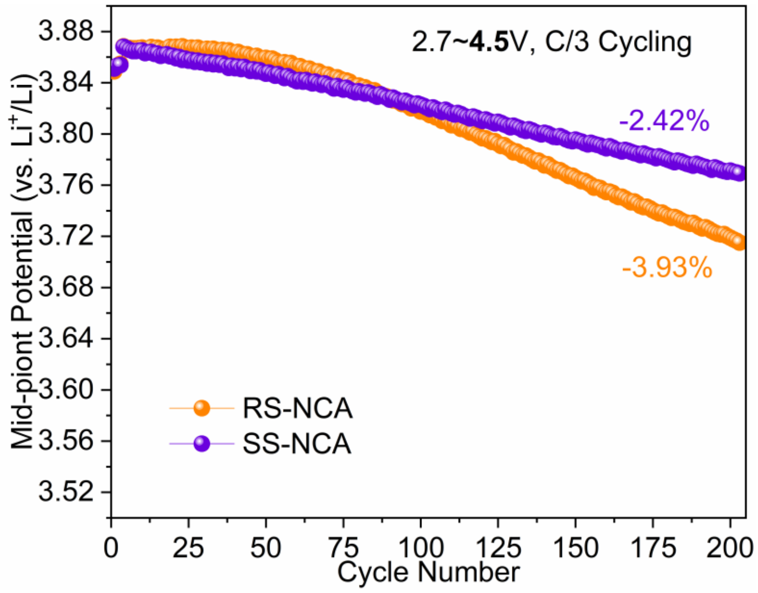

Figure S12. Evolution of discharge mid-point potential of RS-NCA and SS-NCA cathodes during C/3 cycling between 2.7 – 4.5 V.

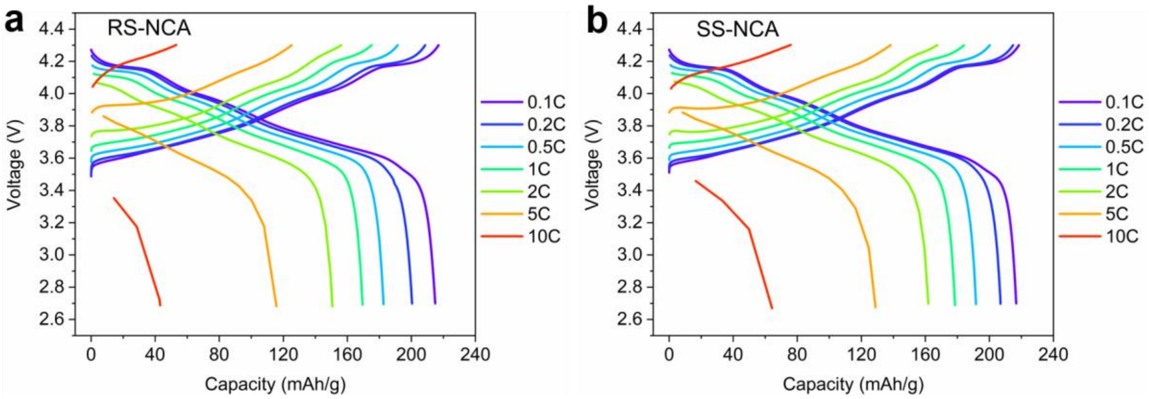

Figure S13. Charge and discharge curves at various rates (0.1, 0.2, 0.5, 1, 2, 5, and 10 C) of RS-NCA (a) and SS-NCA (b) cathodes tested within voltage range of 2.7–4.3 V.

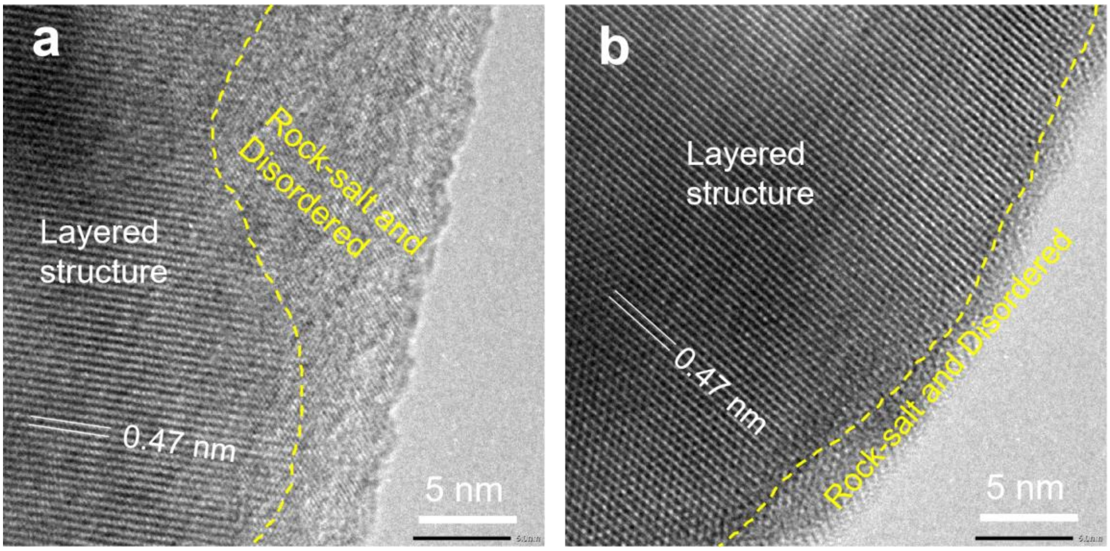

Figure S14. HRTEM images of the grain surface of RS-NCA (a) and SS-NCA (b) cathodes after 200 cycles between 2.7–4.5 V at C/3.

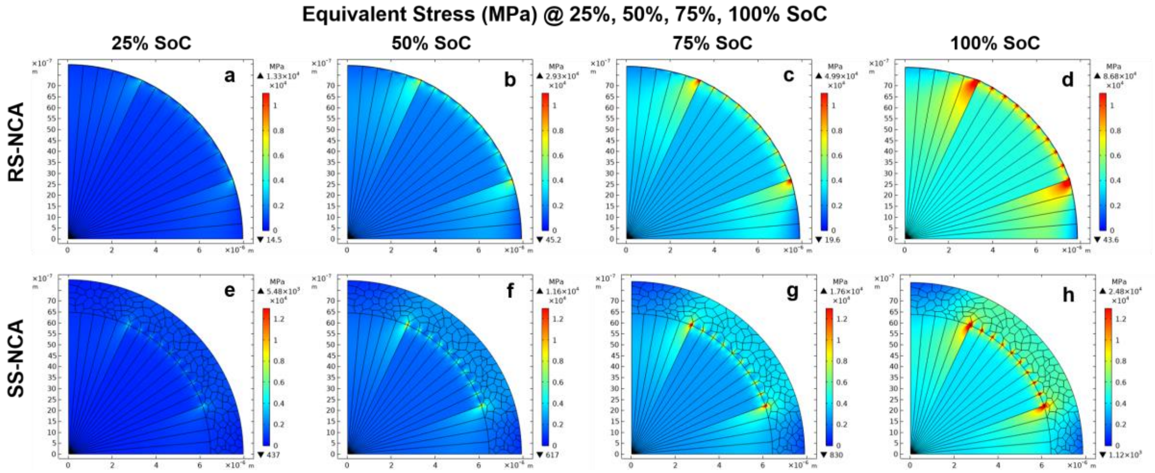

Figure S15. Finite element simulation of the equivalent stress distribution in the cathode particles of RS-NCA (a–d) and SS-NCA (e–h) at various states of charge: 25% SoC, 50% SoC, 75% SoC, and 100% SoC, respectively.

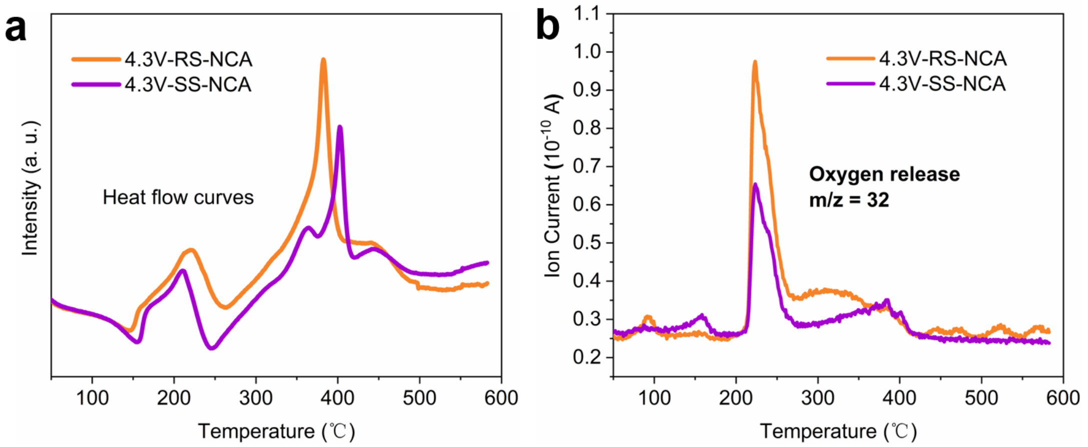

Figure S16. Measurements of oxygen release for charged cathodes by differential scanning calorimetry and mass spectroscopy (DSC-MS). DSC heat flow profile (a) and corresponding oxygen release (b) of the RS-NCA and SS-NCA cathodes charged at 4.3 V.

Table S1. EBSD test settings.

Accelerating Voltage	12.00 kV
Specimen Tilt (degrees)	70.00 °
Hit Rate	80.36 %
Speed of Acquisition	41.42 Hz

Table S2. EBSD phases for acquisition.

Phase	a	b	c	Alpha	Beta	Gamma	Space Group	Database
$Li_{0.99}NiO_2$	2.88 Å	2.88 Å	14.20 Å	90.0 °	90.0 °	120.0 °	166	ICSD

Table S3. Structural parameters determined by Rietveld refinement on the in situ X-ray diffraction patterns of RS-NCA and SS-NCA cathodes during charge/discharge within 2.7–4.3 V at C/10.

**Formula : $LiNi_{0.90}Co_{0.08}Al_{0.02}O_2$ Space group : *R*-3*m***		**Lattice parameters (Rietveld refinement)**				
		Initial state	Fully-charged state	Discharged state after 1 cycle	Lattice strains after fully-charged	Lattice strains after 1 cycle
**RS-NCA cathode**	***a*** /Å	2.876	2.815	2.868	-2.12%	-0.28%
	c /Å	14.272	13.902	14.331	-2.59%	0.41%
	**Volume**/$Å^3$	102.27	95.47	102.13	-6.66%	-0.14%
**SS-NCA cathode**	***a*** /Å	2.871	2.809	2.864	-2.16%	-0.24%
	c /Å	14.209	13.865	14.23	-2.42%	0.15%
	**Volume**/$Å^3$	101.35	94.76	101.26	-6.50%	-0.09%
