# Supplementary figures and images for: Enabling Electrochemical–Mechanical Robustness of Ultra‐High Ni Cathode via Self‐Supported Primary‐Grain‐Alignment Strategy
Source: Adv Sci (Weinh). 2023 Oct 26;10(36):2306347. doi: 10.1002/advs.202306347 (PMC10754075; doi:10.1002/advs.202306347)

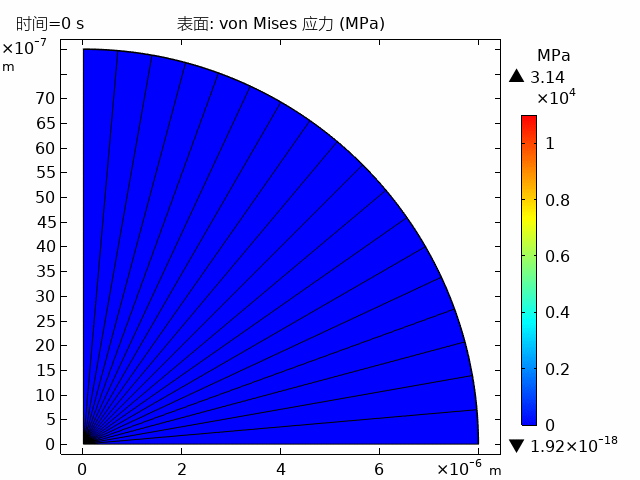

Supplement: Supplementary file 4 — Supplemental Movie S3 [file ADVS-10-2306347-s005.gif]

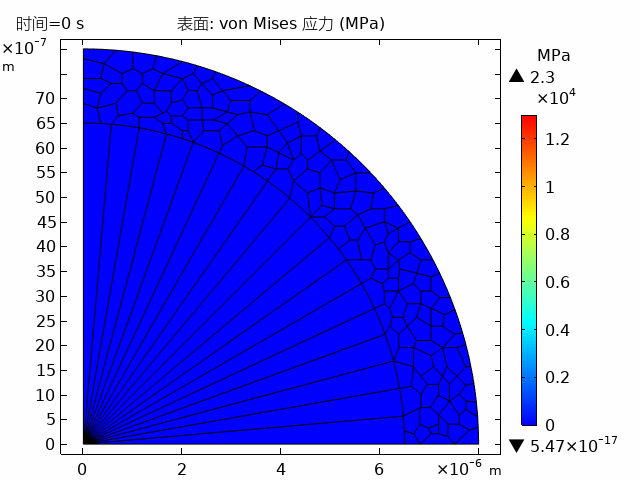

Supplement: Supplementary file 5 — Supplemental Movie S4 [file ADVS-10-2306347-s002.gif]
